# Volume-accumulated reflectivity of the outer retina (integral) on spectral domain optical coherence tomography as a predictor of cone cell density: a pilot study

**DOI:** 10.1186/s12886-023-02827-2

**Published:** 2023-03-14

**Authors:** Wenting Li, Wenwen Chen, Xiyue Zhou, Tingting Jiang, Juan Zhang, Min Wang, Jihong Wu, Junxiang Gu, Qing Chang

**Affiliations:** 1 Department of Ophthalmology, Eye and ENT Hospital of Fudan University, Fudan University, Xuhui District, 83 Fenyang Rd, Shanghai, China; 2 Shanghai Key Laboratory of Visual Impairment and Restoration, Eye, ENT Hospital of Fudan University, Fudan University, Shanghai, China; 3 Key NHC Laboratory of Myopia, Key Laboratory of Myopia, Fudan University), Chinese Academy of Medical Sciences, Shanghai, China

**Keywords:** Spectral domain optical coherence tomography, Adaptive optics, Ellipsoid zone, Interdigitation zone, Inherited retinal diseases

## Abstract

**Background:**

The study aims to investigate the relationship between the volume-accumulated reflectivity (termed “integral”) on spectral domain optical coherence tomography (SD-OCT) and cone density on adaptive optics (AO) imaging.

**Methods:**

In this cross-sectional study, both eyes of 32 healthy subjects and 5 patients with inherited retinal diseases (IRD) were studied. The parameter, integral, was defined as the volume-accumulated reflectivity values in a selected region on OCT images; integrals of the ellipsoid zone (EZ) and interdigitation zone (IZ) were measured at 2°, 3°, 4°, 5°and 6° eccentricity along the four meridians on fovea-centered OCT B-scans. Cone density in the same region was measured using a flood illumination adaptive optics camera RTX1.

**Results:**

Integrals of EZ, IZ and cone density shared similar distribution patterns. Integral of the IZ was better correlated with cone density in both healthy people (r = 0.968, *p* < 0.001) and those with IRD (r = 0.823, *p* < 0.001) than direct measurements of reflectivity on OCT images. A strong correlation was found between best corrected visual acuity (BCVA) and cone density at 2° eccentricity (r = -0.857, *p* = 0.002). BCVA was also correlated with the integral of the IZ at the foveola (r = -0.746, *p* = 0.013) and fovea (r = -0.822, *p* = 0.004).

**Conclusions:**

The new parameter “integral” of the photoreceptor outer segment measured from SD-OCT was noted to correlate with cone density and visual function in this pilot study.

## Introduction

Optical coherence tomography (OCT) is a non-invasive imaging modality providing the morphological features of the retina at all levels with high scanning speed and axial resolution. Since its introduction to ophthalmology, spectral domain OCT (SD-OCT) has been widely used for disease diagnosis, treatment monitoring, and prognosis assessment [1, 2]. The four hyperreflective bands presented in the outer layer of the retina on OCT images from the inner layer to the outer layer were named external limiting membrane (ELM), ellipsoid zone (EZ), interdigitation zone (IZ), and retinal pigment epithelium/Bruch’s complex (RPE), respectively [3]. The segment from EZ to IZ represents the outer segment of the photoreceptor and is found to be initially disrupted in many inherited retinal diseases (IRD) [4].

Several methods for quantifying the outer layers of the retina on OCT have been found to achieve a refined clinical analysis, including the individual band or central macula thickness [5–7], volume [5, 8, 9], disruption length [10, 11] and reflectance [10, 12]. Several studies have been devoted to assessing the correlation between these quantitative values with the visual function, such as best corrected visual acuity (BCVA) and ERG (Electroretinogram) findings [5, 6, 10–14]. Besides, adaptive optics (AO) imaging is a new imaging modality. Several aberrations such as media opacity and prominent vessel shadowing [15] affecting OCT imaging quality were corrected in AO. With a lateral resolution of fewer than 2 microns, AO allows in-vivo visualization of retinal cells and enables the monitoring of a single photoreceptor cell [16, 17]. However, few investigators underlined the association between quantitative assessment on OCT and cone density on AO imaging [18–20]. Although AO provides direct information about cone cells, it is relatively difficult to operate and requires better coordination and fixation. OCT is more widely available, so there is still a need to find a closely related metric to AO in OCT for a quick and accurate assessment of cone density.

Our previous study introduced a new parameter, “integral”, as a quantitative method for the photoreceptor outer segment on OCT [21]. In this study, we further assessed the distribution characteristics of the integral and evaluated its association with cone density measured by adaptive optics imaging.

## Methods

The single-centered cross-sectional study was conducted in Eye and ENT Hospital of Fudan University (Shanghai, China) conformed to the Declaration of Helsinki. The protocol was approved by the Institutional Review Board of the Eye and ENT Hospital of Fudan University. Informed consent was obtained from all participants.

### Participants

Healthy volunteers and patients diagnosed with IRD presenting with outer retinal impairment on OCT B-scans were studied. Healthy subjects from 20 to 39 years were included if the best corrected visual acuity (BCVA) was 0.00LogMAR or better, the refractive error was between -6D and + 3D, and axial length ranged from 22 to 26 mm. All were divided into two age groups, the younger group (< 30 years) and the elder group (≥ 30 years). Subjects with other ocular conditions, media opacities, posterior scleral staphyloma, history of eye surgery or trauma were excluded. Patients who visited the Eye and ENT Hospital of Fudan University between January 2019 and September 2020 and were diagnosed with IRD featuring disruption of the outer retinal layer on OCT cross-sectional B-scans were enrolled. The diagnosis was based on the inheritance pattern, fundus appearance, characteristic electroretinograms or genetic analysis. Exclusion criteria included poor fixation for a fovea-centered OCT or AO scan, poor image quality, media opacities, macular edema, posterior sclera staphyloma, and uncontrolled eye movement.

All subjects underwent a complete eye examination, including slit-lamp examination, fundus imaging, measurement of axial length, spherical error, and BCVA. Ocular axial length was measured by IOL Master 500 (Carl Zeiss Meditec, Dublin, CA, USA). OCT images were collected using SD-OCT (Spectralis HRA + OCT, Heidelberg Engineering, Heidelberg, Germany). Besides, all the patients were performed electrophysiological (ERG) studies and the molecular testing previously reported [22], which included targeted exon sequencing followed by sanger sequencing and segregation analysis.

### Adaptive optics imaging and analysis

**Fig. 1:**
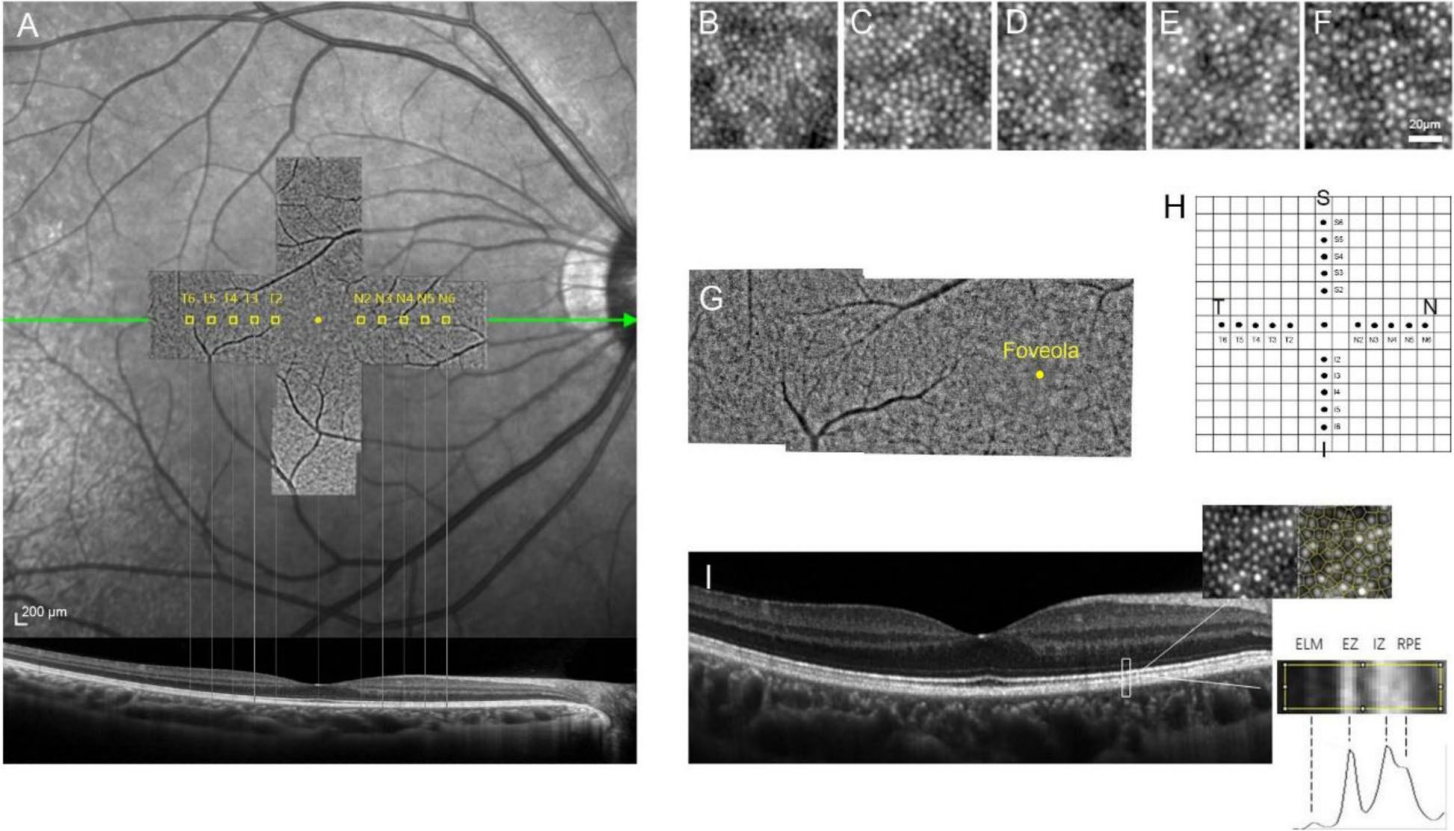
The AO and OCT Images of the right eye in a healthy subject. **A**: Correspondence of the areas captured in the horizontal meridian on the montaged near-infrared retinal fundus image, OCT B-scan and AO montage. Scale bar, 200 μm. **B**-**F**: AO images were acquired at 2°, 3°, 4°, 5°and 6° eccentricity in the horizontal meridian from left to right. Scale bar, 20 μm. **G**: The wide-field AO montage of the AO images in the horizontal meridian created using the montage tool I2K Retina software. **H**: The grid diagram showed all the sampling areas in the four meridians. Each grid on the diagram represented the range of 1°$\times$ 1°. The sampling areas include multiple anatomical regions of the retina (foveola, fovea, parafovea and perifovea). **I**: For each sampling area, cone density and integrals of the hyperreflective layers were measured. The four peaks on the grayscale curve represented ELM, EZ, IZ and RPE-Bruch’s complex from left to right. Abbreviation: AO, adaptive optics; OCT, optical coherence tomography; ELM, external limiting membrane; EZ, ellipsoid zone; IZ, interdigitation zone; RPE, retinal pigment epithelium

AO images were obtained in all the included eyes using a flood illumination (FIO) adaptive optics camera (RTX1, Imagine Eyes, Orsay, France) without pupil dilation. The system was based on a central wavelength of 850 nm. Before image acquisition, the participant’s axial length and the refractive error should be entered to correct for spherical ametropia through an inbuilt formula. A built-in fixation target displayed as a yellow cross was set for participants, first in the central macular, then to a predetermined location in the periphery of the retinal coordinate. Imaging depth was adjusted from 0-90 μm to achieve the sharpest photoreceptor cells. Cone cells within 2 degrees eccentricity could not be accurately identified due to the limitation of device resolution and the effect of macular cell bulging [23]. Thus, images were acquired at 2°, 3°, 4°, 5°and 6°eccentricity along four meridians (superior, inferior, temporal and nasal). Eccentricity was defined as the distance between the foveal center and the captured image center. Each captured image was 4°$\times$ 4° (1200 μm $\times$ 1200 μm) and the final output image was the average of 40 high-resolution raw frames. Good fixation allowed the AO to capture images with the imaging center right at the pre-set location to maximize repeatability. A wide-field AO montage was then created using the montage tool I2K Retina software (Dual Align, Clifton Park, NY, USA) (Fig. 1).

**Fig. 2:**
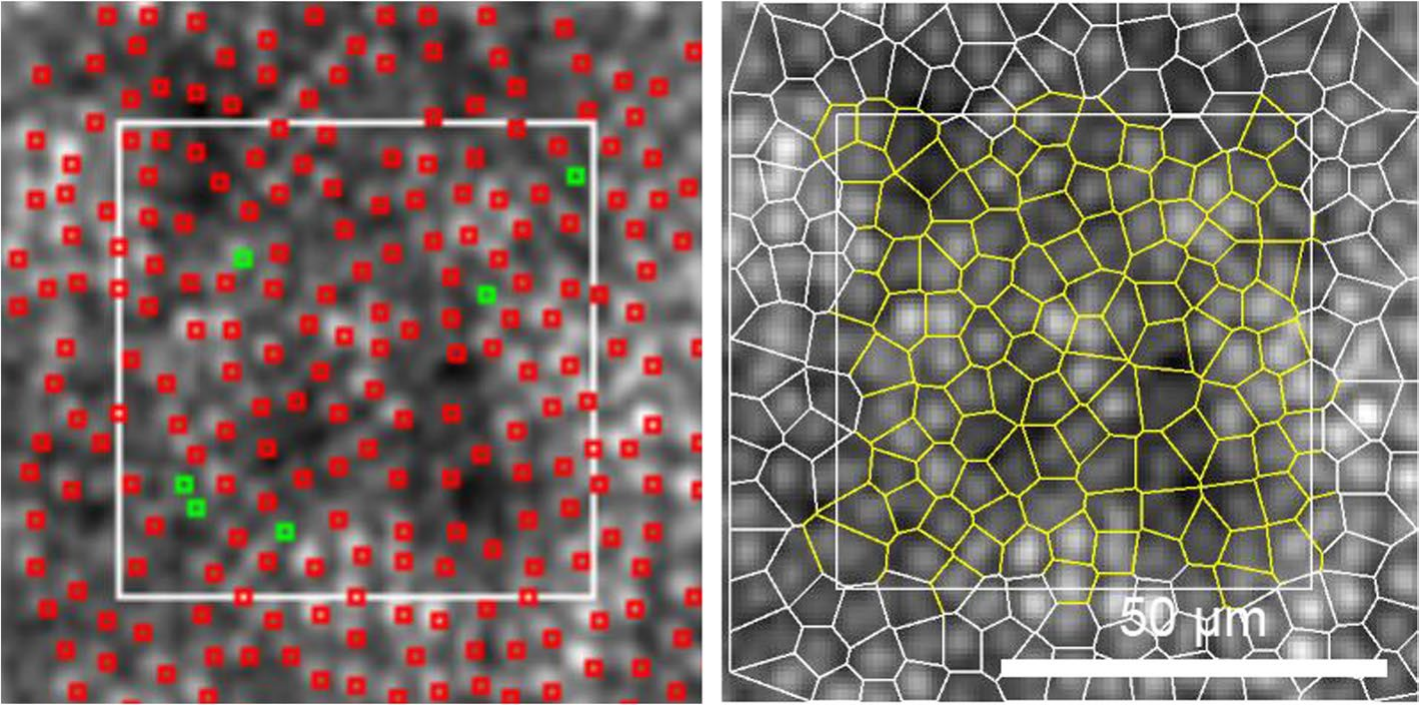
The AO cone recognition in a healthy subject at 2 ^o^ eccentricity. The white square was a 0.3 ^o^ $\times$ 0.3 ^o^(90 μm $\times$ 90 μm) ROI. Left: Red dots represented the cone cells identified by the automated counting software AO detect. Green dots represented the cone cells missed by the automatic recognition. Right: Voronoi tessellation after manual adjustment. Scale bar, 50 μm Abbreviation AO, Adaptive optics

For AO analysis, a 0.3°$\times$ 0.3°(90 μm $\times$ 90 μm) region of interest (ROI) was placed at each eccentricity along the four meridians. ROI was manually shifted slightly away to get a clear cone mosaic image when the ROI fell right at the blood vessels or in the shadows. Cones were identified by an automated counting software (AO detect, Imagine Eyes, France) with reflectance values higher than the surrounding background value. Output results included cone density, cone spacing, and percentage of cones with six neighbors (as determined from the Voronoi diagram). The manual adjustment was made for the cells that were not identified or incorrectly identified in the automatic count (Fig. 2). Cone cell identification was verified by two independent investigators (WL, XZ), and the final presented results were the average of the two measurements.

### Quantitative analysis of photoreceptor outer segment

OCT scan was localized to a 30-degree range centered on the fovea, with horizontal and vertical line scans right across the fovea and a signal-to-noise ratio of no less than 30 dB. A hundred B-scans were averaged for each image by ART (Automatic Real-Time) software. Regions of interest were set at the fovea center and 2°, 3°, 4°, 5°and 6° eccentricity along the four meridians corresponding to the AO sampling areas. Each ROI was 200 μm in width along the scanning line and contained the four hyperreflective bands in the outer retina (Fig. 1).

**Fig. 3:**
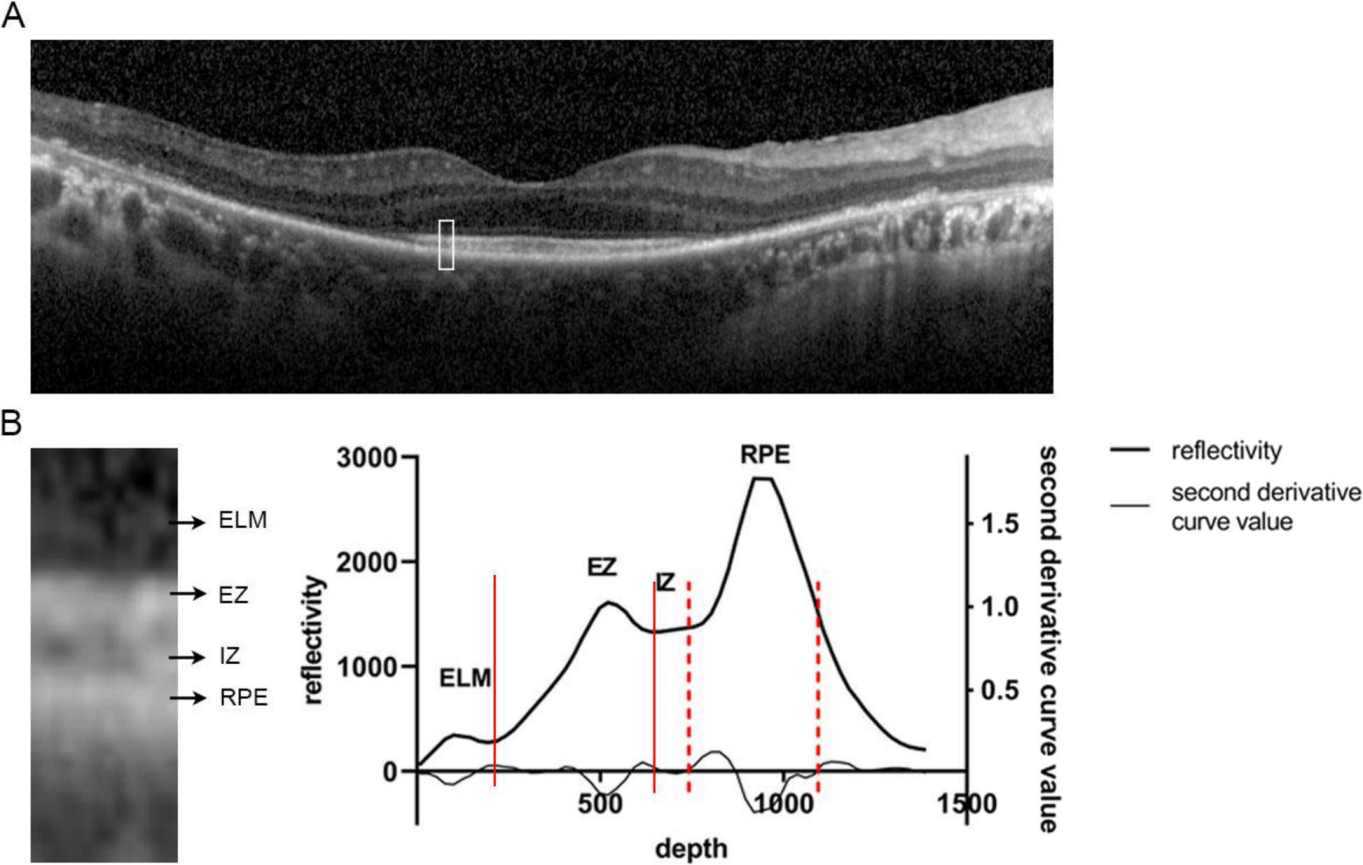
Calculation of integrals through OCT B-scan of the outer retina. Images are of the right eye of a 52-year-old man with retinitis pigmentosa. **A**: The B-scan image of the horizontal line through the fovea. **B**: The sampling area was outlined in white in (A). The high-reflection bands from superficial to deep represent ELM, EZ, IZ and RPE, respectively. The AUC of each peak divided was recorded as the original integral of each band. The integral value was calculated as the percentage ratio of each original integral over the total integral of the four bands at the same sampling position. Abbreviation: OCT, optical coherence tomography; ELM, external limiting membrane; EZ, ellipsoid zone; IZ, interdigitation zone; RPE, retinal pigment epithelium; AUC, area under the curve

The grayscale images were imported to ImageJ (http://imageJ.nih.gov/ij/; National Institutes of Health, Bethesda, MD, USA). Average grayscale values of each row of pixels at each sampling area were calculated and the grayscale curve was plotted (Fig. 3). The calculation of “integral” has been introduced in our previous article [21]. Briefly, the integral of each layer corresponding to the curve peak was accumulated and adjusted using an integration algorithm. The derivation and the second order derivatives of curves could be used to differentiate between adjacent layers in exceptional cases (Fig. 3).

Another currently-used measurement of the outer retina in SD-OCT images, reflectivity, was also acquired by directly measuring the peak greyscale value of each band in the OCT images [18]. The reflectivity value of each band was also adjusted for its percentage over the total value of the four bands.

### Statistical analysis

Statistical analyses were performed using SPSS version 21.0 (SPSS Inc., Chicago, IL, USA).

All the normally-distributed variables were expressed as mean ± standard deviation. BCVA was represented as the logarithm of the minimum angle of resolution (LogMAR). Shapiro–Wilk tests were performed for variable normality. The interocular variability of cone density and integral values was calculated using Mann–Whitney U test. T-tests were performed to compare the integral of each layer and AO results between sex groups, age groups, and the four meridians. After adjustment of age and gender, the correlation of the EZ and IZ integral values in healthy subjects with cone density was assessed respectively using Spearman correlation. A generalized estimating equation (GEE) model was enrolled to adjust the weight of each eye in the statistics considering the potential correlation between the two eyes. Stepwise multiple linear regression was used to analyze the correlation among cone density, BCVA, and integral values of EZ and IZ respectively in IRD patients after adjustment of age and gender. The correlations of integral and reflectivity with cone density were compared using the Z test. Correlation curves were plotted by the commercially available software GraphPad Prism 8 (GraphPad Software, San Diego, CA, USA). P values less than 0.05 were considered statistically significant.

## Results

Sixty-four eyes of 32 healthy subjects (9 males and 23 females aged 29.06 ± 4.51 years, range: 23–39 years) and 10 eyes of 5 IRD patients (1 male and 4 females) were included. Each patient came from a different family. Among the healthy subjects, 18 belonged to the younger group (age < 30y) and 14 belonged to the elder group (age ≥ 30y). All the IRD patients were bilaterally involved, of which one was diagnosed with cone-rod dystrophy (CORD) and four were diagnosed with retinitis pigmentosa (RP). Characteristics of the healthy subjects and IRD patients are shown in Tables 1 and 2, respectively.

**Table 1:**
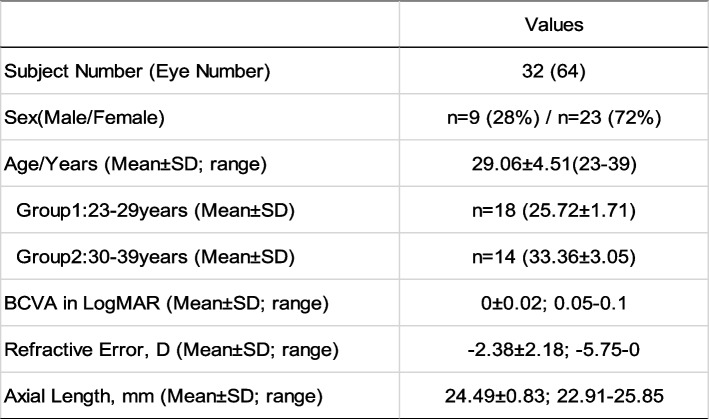
Demographic data of the healthy subjects

Abbreviation: *BCVA* best corrected visual acuity, *SD* standard deviation

**Table 2:**
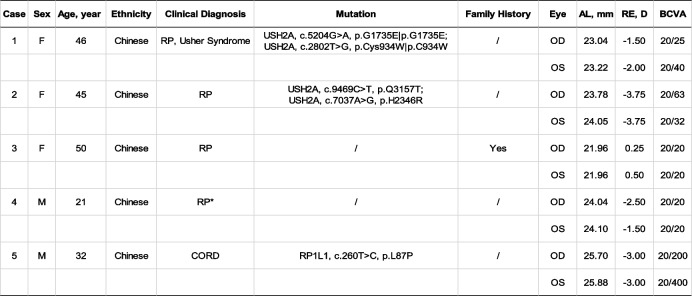
Clinical and genetic characteristics of the 5 IRD patients

*The diagnosis was based on family history, typical symptoms, OCT and visual field findings. Abbreviation: *AL* axial length, *RE* rarefactive error, *BCVA* best corrected visual acuity, *CORD* cone-rod dystrophy, *RP* retinitis pigmentosa

### Distribution characteristics of adaptive optics result in healthy people

**Fig. 4:**
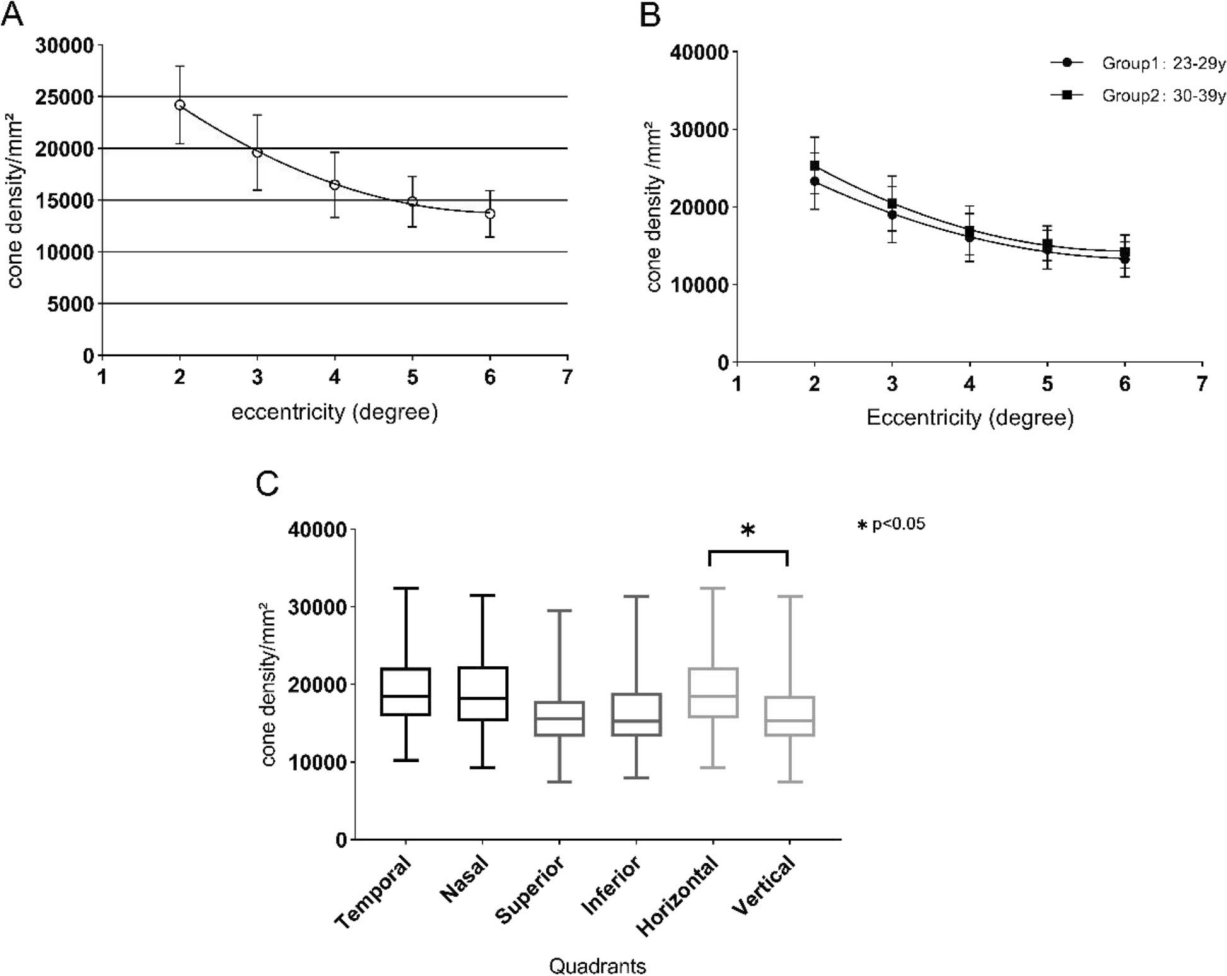
Distribution characteristics of cone density in healthy subjects. **A**: Cone density as a function of eccentricity, decreasing from the fovea to the peripheral retina. **B**: Cone density comparison between younger subjects and elder subjects (group 1, younger group: 23–29 years; group 2, elder group: 30–39 years). Fovea density was higher in the younger subjects at 2° and 6 ^o^ eccentricity (T test, *p* = 0.014 and *p* = 0.006, respectively). **C**: Box plots presenting cone density distribution in the four meridians. Lower cone density was observed in the vertical (superior + inferior) meridian (*p* < 0.05)

Cone density was symmetrical bilateral (Mann–Whitney U test, p = 0.84) and normally distributed at each eccentricity Table 3. No significant difference was found between males and females in each eccentricity. The inter-individual coefficient of variation was on average 17.4%. In both age groups, cone cells showed the same pattern of decline with increasing eccentricity. Fovea density was higher in younger than elder at the fovea (2°) and 6° (T-test, *p* = 0.014 and *p* = 0.006, respectively) but had no difference in other regions (T-test, *p* = 0.146, *p* = 0.432 and *p* = 0.095, from 3° to 5°, respectively) (Fig. 4).

**Table 3:**
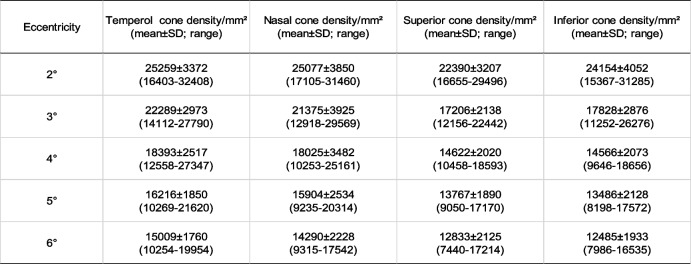
Cone density (/mm2) of the four meridians after manual adjustment and normality test in 64 healthy eyes from 2 to 6 degrees eccentricity

Abbreviation: *SD* Standard deviation

To evaluate the distribution of the cones in different directions, the cone density on the four meridians in both eyes was accumulated and compared using paired T-test. No statistically significant difference in cone density was observed between the nasal and temporal meridians (*p* = 0.78) or the superior and inferior meridians (*p* = 0.99). Further comparison between the vertical and horizontal directions indicated an overall lower cone density on the vertical meridian than on the horizontal meridian (*p* < 0.05) (Fig. 4).

### Macular cone density in patients with inherited retinal diseases

**Fig. 5:**
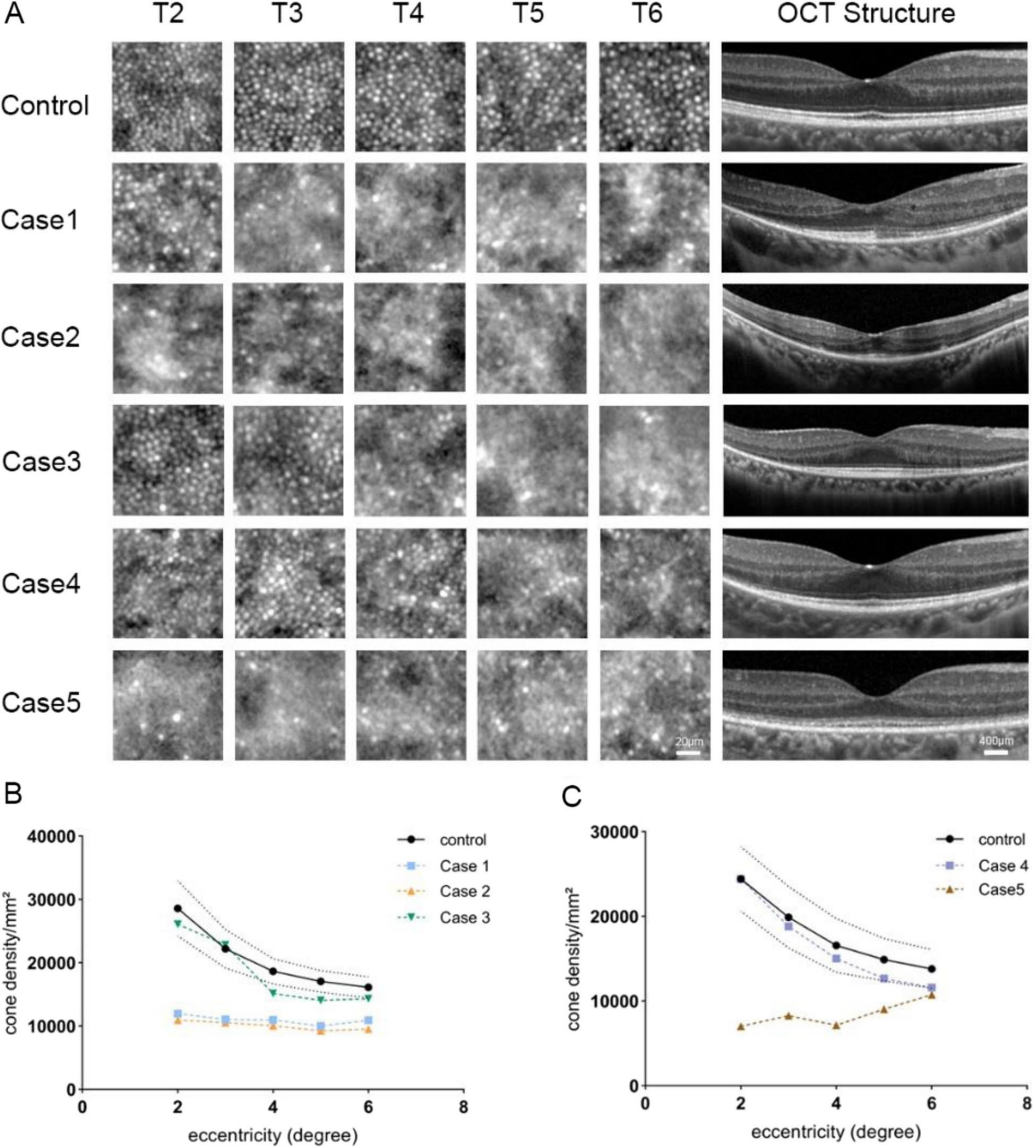
Cone morphology in the temporal meridian in IRD subjects. **A**: Cone morphology and OCT images in IRD subjects and healthy control. Scale bar, AO image: 20 μm; OCT image: 400 μm. **B**/**C**: Comparison of cone density at each eccentricity between cases and healthy control. Healthy control was set as the healthy subjects of the corresponding age group. 95% limits of agreement (LOA) were calculated for comparisons, shown as the dashed lines paralleled with the control group

Figure 5 shows the cone cell morphology and corresponding OCT images in the right eye of the 5 IRD patients. All the patients had ELM preserved with EZ and IZ interruption in the patients with RP, IZ loss and blurred EZ in the patient with CORD. ERG cone responses showed varying degrees of significantly diminished amplitudes and delayed implicit time in all the patients with poor visual acuity. Case 1 and case 2 had significant bilateral visual impairment and loss of typical cone cell mosaic, presenting a decreased density from the foveal to the perifoveal. Case 3 and case 4 had normal BCVA bilaterally with continuous EZ and IZ in the central fovea on OCT images; the AO images showed a typical decreasing cone cell density pattern from the fovea to the peripheral retina. Cone cell density in case 3 was within the normal range at 2° and 3° eccentricity but lower than the normal lower limit at 4° to 6° eccentricity, as shown in Table 3. In case 4, cone density was within the 95% confidential intervals (95% Cis) of normal subjects at all eccentricities closer to the lower limit of the normal range as it progressed to the periphery. All the RP patients showed the progression of photoreceptor degeneration from peripheral to central macula in AO images. The fovea-centered decline of cone density was observed in the patient with CORD (case 5) while the cell mosaic was close to normal in the peripheral macula.

### Distribution characteristics of integral values in healthy people

Integrals in healthy people had mostly consistent distribution characteristics with those of cone density. The average integrals of EZ and IZ were normally distributed (Table 4). Integrals were not significantly different bilaterally (Mann–Whitney U test, *p* = 0.97 and *p* = 0.053, respectively). Gender did not lead to distinctive differences in integral values at the parafoveal and perifoveal (T test, EZ: *p* = 0.347, *p* = 0.092, *p* = 0.205, *p* = 0.643, IZ: *p* = 0.074, *p* = 0.310, *p* = 0.091 and *p* = 0.121, from 3 to 6 ^o^ respectively). However, the IZ integral showed gender difference at 2 degrees (T test, *p* = 0.023), where the EZ integral was of no difference (T test, *p* = 0.141). The EZ and IZ integrals between the two age groups had almost no difference, and the difference was evident only in the EZ integral at the fovea (2°) (T test, *p* = 0.033). For the comparison of integrals among meridians, the difference was observed only for the EZ integral between superior and inferior (T test, *p* = 0.014) Table 5.

**Table 4:**
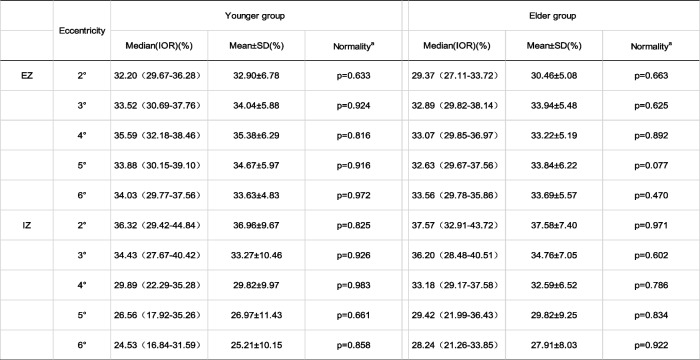
EZ and IZ integrals and normality test in healthy subjects from 2 to 6 degrees eccentricity

^a ^Shapiro-Wilk test. Abbreviation: *EZ*  Ellipsoid zone, *IZ* Interdigitation zone, *SD* Standard deviation

**Table 5:**
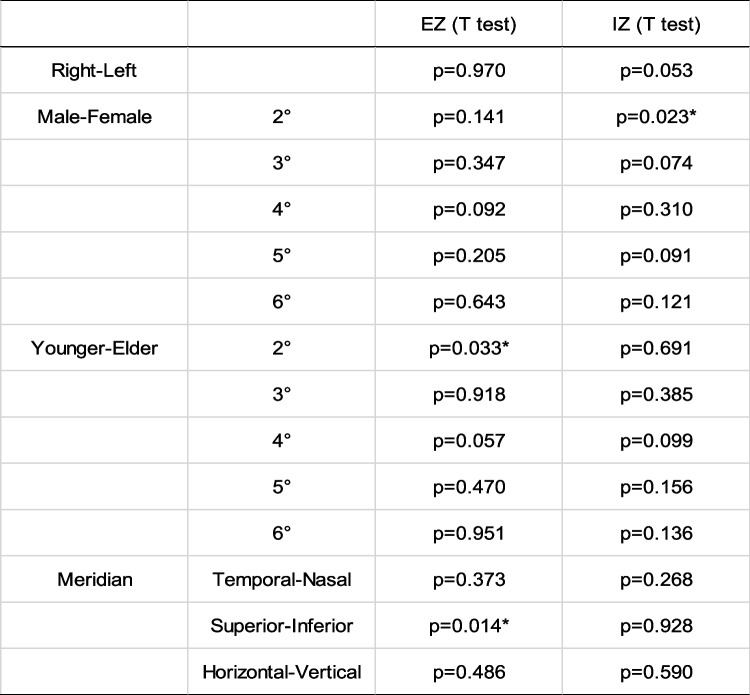
Comparison of cone density (/mm2) for different parameters (laterality, sex, age and meridian)

*p < 0.05. Abbreviation: *EZ* Ellipsoid zone, *IZ* Interdigitation zone

### Relationships among integral values, cone density and BCVA

**Fig. 6:**
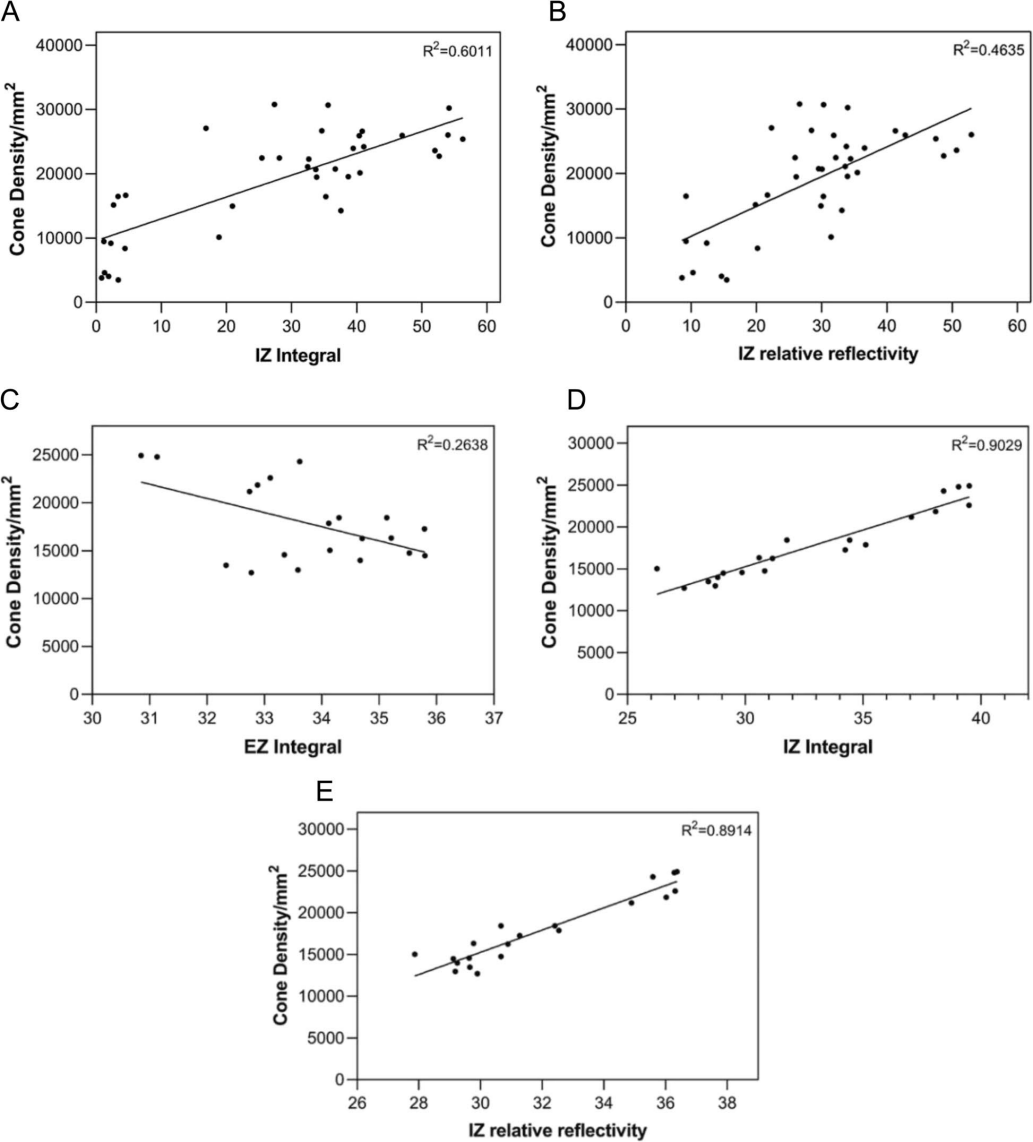
Relationship between the integral, reflectivity and cone density in patients and healthy subjects. Relationship between the IZ integral (A), IZ reflectivity (B) and cone density(/mm^2^) in eyes with inherited retinal diseases (r = 0.823, *p* < 0.001; r = 0.789, *p* < 0.001, respectively), and between the EZ integral (C), IZ integral (D), IZ reflectivity (E) and cone density(/mm^2^) in healthy subjects (r = -0.616, *p* < 0.05; r = 0.968, *p* < 0.001; r = 0.960, *p* < 0.001, respectively) Abbreviation EZ, ellipsoid zone; IZ, interdigitation zone.

After correcting for meridian effects with multiple linear regression, cone cell density was highly corrected with IZ integral (r = 0.968, *p* < 0.001) and IZ reflectivity (r = 0.960, *p* < 0.001). The correlation between cone cell density and IZ integral was significantly higher than that with IZ reflectivity (z = 7.5763, *p* < 0.001). Cone cell density was negatively correlated with EZ integral (r = -0.616, *p* < 0.05) but was not statistically correlated with EZ reflectivity. (Fig. 6).

**Fig. 7:**
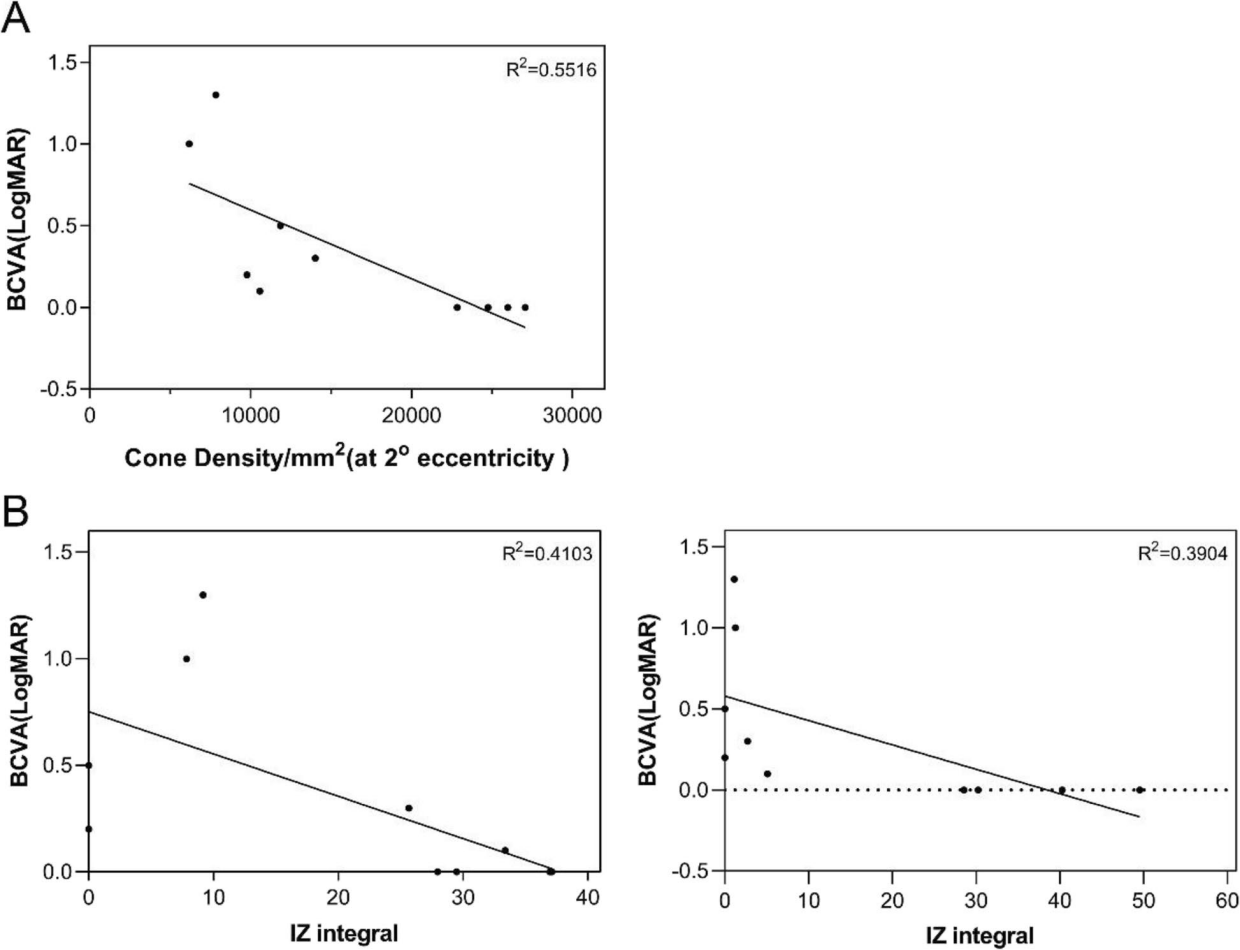
Relationships among integral values, cone density and BCVA. **A**: Relationship between BCVA(LogMAR) and cone density(/mm^2^) (r = -0.857, *p* = 0.002). **B**: Relationship between BCVA(LogMAR) and IZ integrals at the foveola (left) (r = -0.746, *p* = 0.013) and at the fovea (right) (r = -0.822, *p* = 0.004). Abbreviation: BCVA, best corrected visual acuity

Thirty-seven regions of interest that could be counted were selected from images of IRD patients for correlation analysis. After correction using the GEE model for correlation between the two eyes, cone density was found to be significantly correlated with the IZ integral (r = 0.823, *p* < 0.001) and IZ reflectivity (r = 0.789, *p* < 0.001) (Fig. 6). No significant difference was found between the two correlations (z = 0.8770, *p* = 0.3805). A strong correlation was found between BCVA and cone density at 2° eccentricity (r = -0.857, *p* = 0.002), and between BCVA and the IZ integral at the foveola (r = -0.746, *p* = 0.013) and the fovea (2°) (r = -0.822, *p* = 0.004) (Fig. 7). No significant correlation was found between BCVA and reflectivity, either in EZ or IZ.

## Discussion

In this article, we investigated the characteristics of a newly proposed parameter “integral” and cone density distribution in healthy subjects and several patients with IRD. Similar distribution characteristics were observed between the EZ and IZ integrals and cone packing density. The integrals of EZ and IZ both had close relationships with cone density, especially for the correlation between IZ integral and cone density, which indicated that the IZ integral had the potential to reflect cone cell density. In addition, we found that loss of cone cells emerged before the decline of BCVA in IRD patients.

We observed a significant variation in cone density between different subjects, and the average coefficient of variation reached 17%. Previous works of literature reported inter-individual variation between 11 and 20% with the variation highest at the fovea [24–27]. The main difference in cone packing density compared to previous studies was in the cone density at 2° eccentricity, of which the possible explanation might lie in the differences between ethnicity, age composition, subject number, ocular dominance, AO devices, and measurement method (e.g., algorithms, manual adjustment, or the ROI size). Besides, we observed a denser cone density in the horizontal meridian in healthy subjects, which corresponded to the elliptical isodensity contours in histological studies, referred to as horizontal cone streaks [27]. The horizontal cone streaks were observed in several studies using AO, whether based on scanning laser ophthalmology (SLO) or FIO [28, 29]. The effect of age on cone cell density was found mainly at the fovea. The result was similar to Song et al. measuring cone density within 0.5 mm from the center of the fovea using AOSLO [30] and Legras et al. at the same 2° eccentricity using RTX1 [24].

The EZ and IZ integrals shared similar distribution characteristics with cone packing density. The EZ and IZ integral differences were observed between the two age groups, indicating that integral also tends to change with age as cone density. The main difference in our results lay in the horizontal meridian and the vertical meridian. Unlike cone density, neither EZ nor IZ integral difference was observed between the horizontal and the vertical meridian. In OCT images, the reflections of EZ and IZ together depend on cone cells’ structure, of which only a single analysis of EZ or IZ cannot fully reflect. We will further synthetically analyze the combined effects of EZ and IZ in future studies.

The comparability was based on a similar imaging principle: the light reflection from a specific structure. Cone recognition in the flood illumination adaptive optics applied in our study is based on an axial boundary of the cone outer segment using coherent interference of two reflections [31], while the main light scattering organelle is mitochondria in the cone cell inner segment on OCT [32]. Previous articles have reported the correlation between OCT reflectivity and cone density [18, 19]. Based on the existing literature, we further included IRD patients, expanded the imaging range, and improved the reflectivity measurement using the integration algorithm. Results showed that the outer retinal lesion in IRD begins with photoreceptor cell degeneration, so the suggestive role of integral is more meaningful early in the lesion. Besides, AO-FIO allows imaging of all healthy cone cells in healthy subjects. In patients, however, shortened cone cells with IZ disruption may not be detected by AO-FIO, resulting in fewer detected cone cells than the actual cone number. Therefore, we measured the correlation between integrals and cone density in healthy individuals and patients separately. We also had an extensive measurement range with 6 ^o^ eccentricity in each meridian.

Several inherited retinal diseases, including retinitis pigmentosa, cone or cone-rod dystrophy, and Stargardt disease, are characterized by progressive disruption of EZ and IZ on OCT. OCT changes can occur even in patients with normal fundus photography or undamaged visual acuity. The IZ’s visible disruption precedes the EZ’s disruption on OCT images [33]. Therefore, early quantification of the outer retina on OCT, especially of the IZ layer in IRD patients, is of great value for early diagnosis and prognostic prediction. Previous OCT quantification methods focused mainly on the thickness or reflectivity of a particular layer. With the introduction of adaptive optics, a cone density decrease was found where the EZ and IZ bands remained continuous on OCT [34, 35]. A previous study has found a better connection between cone density and reflectivity than between cone density and EZ thickness [18]. Thus, calculation concerning reflectivity was a more accurate method than thickness measurement with the additional advantage of reduced errors in manual or automatic segmentation. Our parameter “integral” differed from reflectivity as it calculated the whole hyperreflective substances in a certain layer, taking into account the entire thickness of the layer rather than the peak reflectivity in the layer as in previous studies. In this article limiting patients to those with IRD, we found that the IZ integral was more suggestive of cone cell density than the IZ reflectivity in healthy subjects. In our results, the IZ integral was also significantly correlated with BCVA and cone density at the fovea. All the results suggested that the parameter “integral” measured the cumulative spatial effect of reflectivity and thus served as an improved indicator of the outer retinal structure, which could be a biomarker of cone disruption in IRD patients at early stages.

There were several limitations in our study. We included a healthy population aged 20 to 40 years, which did not match the age of the majority of our patients. The number of patients we included was small and further experiments were needed for more genotype-specific patients to analyze the phenotype-genotype correlation. Furthermore, cone cells with IZ disruption could not be detected by AO-FIO, which might further require split-detection adaptive optics, a non-confocal AOSLO, to detect these cone cells by simultaneously recording signals from the photoreceptor inner segment [35, 36]. Besides, good fixation was challenging for patients with poor visual conditions. Despite the good cooperativeness of our included subjects to reduce motion artifacts, the repeatability of AO imaging was still affected by the narrow imaging range and the asthenopia associated with prolonged gazing at the light spot. In addition, the slope of the peripheral retina affected the Stiles-Crawford properties of the photoreceptors in AO imaging and also affected the integral calculation in OCT [37]. We calculated the average of multiple regions to minimize its effect. Finally, the study was a preliminary research and prospective studies were needed to verify the feasibility of our method.

In conclusion, the parameter “integral” measured in OCT images could be a feasible estimator of cone density if the AO devices were unavailable. EZ and IZ Integrals have the potential to be applied to early detection, function prediction, and longitudinal follow-up of more photoreceptor-involved diseases.

## Data Availability

The datasets used and/or analyzed during the current study are available from the corresponding authors on reasonable request.

## Abbreviations

SD-OCT: Spectral domain optical coherence tomography
AO: Adaptive optics
IRD: Inherited retinal diseases
EZ: Ellipsoid zone
IZ: Interdigitation zone
OCT: Optical coherence tomography
ELM: External limiting membrane
RPE: Retinal pigment epithelium
BCVA: Best corrected visual acuity
ERG: Electroretinogram
FIO: Flood illumination
ROI: Region of interest
LogMAR: Logarithm of the minimum angle of resolution
GEE: Generalized estimating equation
CORD: Cone-rod dystrophy
RP: Retinitis pigmentosa
SLO: Scanning laser ophthalmology
